# Treatment outcomes of acute poptoperative infectious endophthalmitis

**DOI:** 10.1186/s12886-021-02144-6

**Published:** 2021-10-29

**Authors:** Kai-Ling Peng, Ya-Hsin Kung, Hui-Shuang Tsai, Tsung-Tien Wu

**Affiliations:** 1 Department of Ophthalmology, Kaohsiung Veterans General Hospital, 386, Ta-Chung 1st Road, Kaohsiung, 813 Taiwan, R.O.C.; 2 Shu-Zen Junior College of Medicine and Management, Kaohsiung,, Taiwan, R.O.C.; 3 School of Medicine, National Yang Ming Chiao Tung University, Taipei, Taiwan, R.O.C.

**Keywords:** Acute infectious postoperative endophthalmitis, Cataract surgery, Intravitreal injection, Vitrectomy

## Abstract

**Background:**

Acute postoperative endophthalmitis is one of the most severe complications of modern ophthalmic procedures including cataract surgeries, vitrectomy and intravitreal injection (IVI). We evaluated the treatment outcomes of acute postoperative infectious endophthalmitis.

**Methods:**

In this retrospective study, we collected data from 82 patients with acute infectious endophthalmitis within 6 weeks after intraocular surgeries, including cataract surgeries, vitreoretinal surgeries, and IVI, from January 2010 to December 2019. We analyzed the pre-treatment, treatment-related and post-treatment factors that affected visual outcomes.

**Results:**

The mean age was 67.65 ± 9.52 years, the proportion of male patients was 56.1%. The mean baseline vision was 1.92 (Snellen Equivalent SE], counting finger [CF]) ± 0.54 logarithm of the minimum angle of resolution (log MAR) and the mean final vision was 0.71 (SE, 39/200) ± 0.80 logMAR. Visual improvement was significant (*P* < 0.001). The pre-treatment factors affecting final visual outcomes were diabetes, hemodialysis, baseline vision, signs of vitreous opacity, and different surgeries before endophthalmitis; the treatment-related factors affecting visual outcomes were the choice factors between IVI of antibiotics alone and vitrectomy combined with IVI of antibiotics, and the injection numbers of antibiotics; post-treatment factors affecting visual outcomes were complications such as retinal detachment (RD), glaucoma and macular pucker. Furthermore, prior cataract surgery was associated with a better mean final vision of 0.57 (SE, 54/200) ± 0.67 logMAR while prior vitrectomy resulted in the worst mean final vision of 1.38 (SE, 21/500) ± 0.75 logMAR.

**Conclusions:**

The important factors that affected the final visual prognosis, included diabetes, hemodialysis, baseline vision, severity of vitritis, treatment strategies and complications. The treatment outcomes revealed better final vision in prior cataract surgery than vitrectomy.

## Introduction

In 1997, Aaberg et al. [1] published their previous 10-year data on the incidence of postoperative endophthalmitis for all surgical procedures in which the incidence after cataract removal with or without intraocular lens (IOL) was 0.09% (17/18530) and that after pars plana vitrectomy (PPV) was 0.04% (2/4583). This rate doubled to 0.15% in a 1999 report by Schmitz et al.; in 2006, the rate nearly doubled again to 0.265% according to meta-analysis by Taban et al. and to 0.3% according to the European Society of Cataract and Refractive Surgeons (ESCRS) intracameral antibiotic prophylaxis study [1–3]. This upward surge has been attributed to many factors, including the evolution of clear corneal incisions, temporal incisions, use of topical anesthesia and poor wound construction [4]. Studies from the American Academy of Ophthalmology Intelligent Research in Sight (IRIS) Registry which is uniquely suited to study rare ophthalmic conditions and provide insight into real-world practice patterns and outcomes, have revealed that the incidence of acute-onset endophthalmitis after cataract surgery from 2013 to 2017 [5] was approximately 0.04%.

The incidence of endophthalmitis after vitrectomy has been historically low. For scleral and conjunctival sutures, the recent advent of 20-, 23- and 25-gauge (G) trocar systems that self-seal on withdrawal and ensure a sutureless state at the conclusion of the surgery have improved patients’ discomfort, cosmesis, and rehabilitation. Kunnimoto et al. noted a nearly six-fold increase in the number of endophthalmitis cases after 25-G surgery when compared with 20-G sutures; (0.23% vs. 0.04%, respectively) [6]. Vitreous wick formation, retention of greater vitreous gel allowing easier bacterial adhesion, and initial hypotony with wound leaks were postulated as the possible causative factors [4].

Office-based injections of intraocular gas and antibiotics have been the mainstay of ophthalmic therapeutics for retinal detachment (RD) and infection. In recent years, the advent of anti-vascular endothelial growth factors (anti-VEGF) and triamcinolone for the treatment of exudative macular degeneration, retinal vein occlusion and diabetic macular edema has dramatically increased the amount of patient exposures [6, 7]. Although endophthalmitis rates have remained low, Tarragon and colleagues have reported post-injection infection rates of approximately 0.053% in 2016 [8], and the sheer number of IVIs has increased the overall number of cases of postoperative infection [8–10].

Thus, we aimed to evaluate the treatment outcomes of acute infectious postoperative endophthalmitis and further analyze the factors related to final visual outcomes in cases of acute infectious postoperative endophthalmitis.

## Methods

In this retrospective study, the study followed the principles outlined in the Declaration of Helsinki, and the study was approved by the hospital’s Institutional Review Board. We reviewed the medical records of consecutive patients with infectious endophthalmitis after intraocular procedures within 6 weeks between January 2010 and December 2019. The inclusion criteria were as follows: infectious endophthalmitis within 6 weeks after intraocular procedures including cataract surgeries, vitrectomy, and IVI. The exclusion criteria were patients with endogenous endophthalmitis, iritis, uveitis, those who underwent intraocular surgeries more than 6 weeks before the presence of infectious endophthalmitis, those with a history of trauma, and those who were followed up for less than 1 month.

All infected patients underwent an evaluation that recorded detailed information of their latest ocular surgery history along with complete ophthalmological examination at presentation, which included measurements of Snellen best-corrected visual acuity (BCVA), slit-lamp biomicroscopy, external eye and color fundus photography, and indirect ophthalmoscopy. BCVA was measured using Snellen charts, and subsequently converted into logarithm of the minimum angle of resolution (logMAR) values for statistical analysis [11].

We recorded each patient’s baseline demographic and medical information, including age, sex, systemic diseases such as diabetic mellitus (DM), hypertension (HTN), heart disease, renal disease and liver diseases. Data regarding the pretreatment factors, including prior intraocular procedures such as cataract surgeries, vitrectomy, and IVI; the durations between prior intraocular surgeries and symptom flare-up; the interval from symptom flare-up to treatment; symptoms including blurred vision and pain; and baseline vision were also recorded. In addition, we also collected information regarding pretreatment ocular signs involving the anterior segments such as intraocular pressure (IOP), the presence of hypopyon; and the reactions of posterior segments with mild to moderate vitritis, in which the disc and retinal vessels could be seen, or severe vitritis in which the disc and retinal vessels were all occluded from the fundus.

The treatment-related factors that were assessed included positive or negative findings of culture results, IVI of antibiotics alone, or vitrectomy with concomitant IVI of antibiotics, pre-vitrectomy IVI of antibiotics or not, post-vitrectomy IVI of antibiotics or not, and total amounts of IVI of antibiotics and vitrectomy. Before treatment, we collected aqueous or vitreous samples for bacterial culture. For IVI of antibiotics, we administered *ceftazidime* 2.25 mg / 0.1 mL and *vancomycin* 1 mg / 0.1 mL at 3.5–4.0 mm posterior to the corneal limbus. For vitrectomy, the procedures were performed by well-trained retinal specialists. All surgical procedures included the following steps: three-port 20- or 23G PPV, vitreous sample or aqueous sample collection for culture, a core vitrectomy, and concomitant IVI of antibiotics. The three-port 20G PPVs were performed before June 30, 2014, while three-port 23G PPVs were performed after July 1st, 2014.

Furthermore, we also recorded post-treatment factors including post-treatment visions at 1 month, 3 months and final visions, complications such as retinal detachment (RD), glaucoma and macular pucker, and follow-up time.

We used Pearson correlation to compare the final visual outcomes with the continuous variables. For categorical variables, we used an independent *t*-test to compare the differences in the final visual outcomes. We further divided the participants based on the prior surgeries including cataract surgery, vitrectomy, and IVI. For continuous variables, we used an analysis of variance. For categorical variables, a chi-square test was used. Data were analyzed using IBM SPSS statistical software version 20.0 (Armonk, NY: IBM Corp). A *P* value < 0.05 was considered significant.

## Results

During the study period, 82 patients who had undergone surgeries and subsequently developed infectious endophthalmitis met the inclusion criteria. Men accounted for 56.10% (46/82) of the patients, and 35.4% (30/82) of the cases involved the right eye. The mean age was 67.65 ± 9.52 years (range: 43 to 87 years). The mean baseline vision was 1.92 (Snellen Equivalent [SE], counting finger [CF]) ± 0.56 logMAR (range: 0.3–2.3 logMAR) and was significantly different from the final vision (*P* = 0.030). The mean baseline IOP was 16.00 ± 7.62 mmHg (range: 10–27 mmHg) without significance. The mean final vision was 0.71 (SE, 39/200) ± 0.80 logMAR (range: 0.3–2.3 logMAR). The improvement from baseline to final vision was significant (*P* < 0.001). The mean follow-up period was 14.40 ± 18.99 months (median: 4 months). Table 1 summarizes the detailed data on acute postoperative infectious endophthalmitis, including the data of pre-treatment, treatment-related, and post-treatment factors.

**Table 1:** The detailed data on acute postoperative infectious endophthalmitis, including the pre-treatment, treatment-related, and post-treatment factors

	N (%)/Mean (SD)	Final vision (LogMAR)	*P*
**Age,** years	67.65(9.52)		0.374^a^
**Eye(OD)**	29 (35.4)		0.816^b^
**Gender (M)**	46 (56.1)		0.924^b^
**Systemic disease**			
Diabetes	30 (36.60)	0.54 (0.64) / 1.03 (0.96)	0.019^b^*
Hypertension	38 (46.30)		0.412^b^
Heart disease	10 (12.20)		0.603^b^
Hemodialysis	4 (4.90)	0.66 (0.75) / 1.80 (1.00)	0.004^b^*
Liver disease	3 (3.70)		0.836^b^
**Duration,** days	3.26 (3.71)		0.631^a^
**Interval,** days	2.30 (2.08)		0.888^a^
**Symptoms**			
Blur	67 (81.70)		0.669^b^
Pain	48 (58.50)		0.366^b^
**Baseline vision**	1.92 (0.54)		0.030^a^*
**Baseline IOP,** mmHg	16.00 (7.62)		0.051^a^
**Surgery before endophthalmitis**			
cataract	66 (80.49)	0.57 (0.68)	0.001^c^*
VT	12 (14.63)	1.38 (1.07)	
Intravitreal injection	4 (4.88)	0.97 (0.74)	
**Signs**			
Hypopyon	67 (81.70)		0.844^b^
Vitritis(mild to moderate / severe)	8 (8.5) / 75 (91.50)	0.12 (0.11) / 0.76 (0.81)	< 0.001^b^*
**Treatment of endophthalmitis**			
Pre-VT IVI antibiotics	43 (52.4)		0.740^b^
VT combined IVI antibiotics VT with 23 G/20 G	71 (86.60) 46 (50.1)/34 (34.14)	0.78 (0.84) 0.77 (0.85)/0.62 (0.79)	< 0.001^b^* 0.395 ^b^
Post-VT IVI antibiotics	51 (62.20)		0.230^b^
IVI antibiotics without VT	11 (13.40)	0.28 (0.16)	< 0.001^b^*
IVI antibiotics amount	2.59 (1.23)		0.014^a^*
**Culture positive**	30 (36.60)		0.076^b^
**Complications**	18 (22.00)	0.5 (0.63) / 1.44 (0.89)	< 0.001^b^*
Retinal detachment	8 (9.75)	0.65 (0.75) / 1.19 (1.08)	0.214^b^
**Final vision**	0.71 (0.80)		< 0.001^d*^
**Follow-up time**	14.40 (18.99)		0.945^a^

^*^P < 0.05, ^a^Pearson correlation, ^b^ independent *t*-test; ^c^ANOVA; ^d^Paired *t*-test with baseline visions; N., number; %, percentage; G, gauge; VT, vitrectomy; IVI, intravitreal injection; SD, standard deviation; logMAR, logarithm of minimum angle of resolution

### Pre-treatment factors

DM was recorded in 36.60% (30/82) of the patients in this study, and showed a significant effect on final vision (*P* = 0.019), since patients with DM had a worse mean final vision of 1.03 (SE, 37/400) ± 0.96 logMAR than those without DM 0.54 (SE, 58/200) ± 0.63 logMAR. Renal disease was recorded in 4.90% (4/82) of the patients, and it also showed a significant effect on final vision (*P* = 0.004): patients with renal disease had a worse mean final vision of 1.80 (SE, CF) ± 1.00 logMAR than those without renal disease 0.66 (SE, 45/200) ± 0.75 logMAR.

The mean duration from the prior intraocular surgeries to symptoms flare-up was 3.26 ± 3.71 days (median: 2 days). The mean interval from symptom onset to treatment was 2.30 ± 2.08 days (median: 4 days). The symptoms included blurred vision at first presentation in 81.70% (67/82) of the cases and pain in 58.50% (48/82) of the cases.

Among prior intraocular procedures, cataract surgeries accounted for 80.49% (66/82) of the cases, vitrectomy was performed in 14.63% (12/82), and IVI accounted for the remaining 4.88% (4/82). The comparison between cataract surgeries and vitrectomy showed a significant effect on the final visions (*P* = 0.001) since prior cataract surgeries achieved a better mean final vision of 0.57 (SE, 54/200) ± 0.68 logMAR, while prior vitrectomy resulted in the worst mean final vision of 1.38 (SE, 21/500) ± 1.07 logMAR. Patients who received an IVI had a mean final vision of 0.97 (SE, 43/400) ± 0.74 logMAR. Hypopyon signs were observed in 81.70% (68/82) of the patients. Severe vitritis, that obscured the fundus accounted for 91.50% (75/82) of the cases and had a significant effect on the final vision (*P* < 0.001); mild to moderate vitritis in which disc and retinal vessels could be seen yielded a better final vision of 0.12 (SE, 76/100) ± 0.11 log MAR, while severe vitritis that occluded the disc and retinal vessels showed worse final vision of 0.76 (SE, 35/200) ± 0.81 logMAR (*P* < 0.001).

### Treatment-related factors

Among treatment-related factors, vitrectomy combined with IVI of antibiotics accounted for 86.60% (71/82) of the cases, while IVI without vitrectomy accounted for the remaining 13.40% (11/82). Both procedures showed significant association with final vision (*P* < 0.001), since vitrectomy combined with IVI of antibiotics yielded a worse mean final vision of 0.78 (SE, 33/200) ± 0.84 log MAR, while IVI of antibiotics alone achieved a better mean final vision of 0.28 (SE, 53/100) ± 0.16 logMAR. Pre-vitrectomy IVI of antibiotics was performed in 52.40% of the cases (43/82), while post-vitrectomy IVI of antibiotics performed in 62.7% (51/82). The mean number of antibiotic IVIs with concomitant vitrectomy was 2.59 ± 1.23 (range: 1–7), which showed a significant effect on final vision (*P* = 0.014). However, treatment with 20-G (34.14%; 34/82) and 23-G (50.10%; 46/82) vitrectomy did not show a significant effect on final vision.

### Post-treatment factors

Positive results for culture were obtained in 36.60% (30/82) and did not show a significant association with final vision. Complications after treatment had a significant effect on final vision (*P* < 0.001) since patients with complications had a worse mean final vision of 1.44 (SE, 18/500) ± 0.89 log MAR while those without complications achieved a better final vision of 0.5 (SE, 63/200) ± 0.63 logMAR. In total, there were total 18 eyes (22%) with post-treatment complications. Among them, one eye (5.5%, 1/18)) developed a macular pucker, one eye (5.5%, 1/18) developed severe endophthalmitis and needed evisceration at last, two eyes (11.1%, 2/18) developed corneal decompensaiotn, three eyes (16.67%, 3/18) developed macular scar or atrophy, three eyes (16.67%, 3/18) developed glaucoma under anti-glaucomatic medications and with eight eyes (44.44%, 8/18) developed RD. The incidence of RD, one of the post-treatment complications, was 9.75% (8/82), although it did not show a significant effect on final vision. All these eight cases had undergone VT combined IVI as treatment. There was 62.5% (5/8) in the group of prior cataract surgery whose mean final vision varied from hand motion (HM) to 6/20. Of these five cases, one patient developed rhegmatogenous RD (RRD) as a complication and was lost follow-up and, one developed recurrent RD, which attached at last with a poor final vision of CF. Three of them had a final vision ranging from 6/20 to 2/200 with foveal thinning. The remaining cases [37.5% (3/8)] were in the group of prior vitrectomy whose final vision was all worse than HM; the original cause of the disease was proliferative diabetic retinopathy (PDR) with vitreous hemorrhage (VH) in two cases and vitreomacular traction (VMT) in one case that developed recurrent RD later, which finally attached with a poor final vision of CF.

In assessments based on positive cultures, 63.33% (19/30) of the cases showed gram-positive cocci, of which *Staphylococcus aureus* accounted for 23.33% (7/30), *Enterococcus faecalis* accounted for 20.00% (6/30), and *Staphylococcus epidermidis* accounted for 16.67% (5/30) of the cases. In contrast, 30.00% (9/30) of the cases showed gram-negative bacteria which *Pseudomona*s accounted for 16.67% (5/30) with worst mean final vision of 1.22 (SE, 12/200) ± 1.02 logMAR. The cultured bacteria in all culture-positive cases with associated mean final vision are summarized in Table 2.

**Table 2:** The cultured bacteria in all culture-positive cases are listed with associated final vision

Positive culture results	%	***N*** = 30	Final vision (logMAR) Mean (SD)
**Gram positive cocci**	**63.33%**	**19/30**	**0.81 (0.79)**
*Staphylococcus aureus*	23.33%	7/30	0.86 (0.84)
*Enterococcus faecalis*	20.00%	6/30	0.79 (0.80)
*Staphylococcus epidermidis*	16.67%	5/30	0.98 (0.77)
Stapphylococcus hemolytic	3.33%	1/30	
**Gram positive bacteria**	**3.33%**	**1/30**	
**Gram negative bacteria**	**30.00%**	**9/30**	**1.22 (0.97)**
Pseudomonas	16.67%	5/30	1.22 (1.02)
Citrobacter	6.67%	2/30	
**Mixed flora**	**3.33%**	**1/30**	

### Comparison of different prior intraocular surgeries

We further analyzed the differences between different prior intraocular procedures and summarized the findings in Table 3. Sixty six eyes showed cataract-induced endophthalmitis. Twelve eyes showed vitrectomy-induced endophthalmitis, of which one had RRD, two had VMT syndrome, four had macular pucker and five had PDR with VH. Four eyes showed IVI-induced endophthalmitis of which 3 were injected with bevacizumab and 1 received ranibizumab. The mean age in the group that underwent cataract surgery was higher than that in the other groups (69.27 ± 8.04 years; range: 51–87 years), and the difference was significant (*P* = 0.005). The mean duration from the intraocular procedures to symptom flare-up in the group of intravitreal injection was longer than that in the other groups (6.0 ± 5.89 days), and the mean interval between the symptom flare-up and treatment in the IVI group was shorter than that in the other groups (1.75 ± 0.96 days). The rate of DM in patients showing acute postoperative infectious endophthalmitis appeared to be high for all three surgical procedures, which was 33.33% in those who underwent cataract surgeries and 50% in those who underwent vitrectomy and IVI. Signs of hypopyon appeared more frequently (83.33%) in eyes with prior cataract surgeries than in those with prior vitrectomy and IVI. Vitreous opacity appeared more frequently in eyes that had undergone prior vitrectomy and IVI (100%) than in those that had received prior cataract surgeries (89.39%). With respect to treatment strategies, while patients who had previously undergone vitrectomy and IVI were all initially treated by vitrectomy combined with IVI of antibiotics, while those who had received cataract surgery were either treated by vitrectomy (83.33%) or IVI alone (16.66%). The rate of positive cultures was 100% in the IVI group, 41.67% in the vitrectomy group, and 31.8% in the cataract surgery group. The mean baseline vision was worse in the vitrectomy group (2.09 [SE, CF to HM] ± 0.35 logMAR) and the IVI group (2.08 [SE, CF to HM] ± 0.26 logMAR, while the cataract surgery group showed a better mean baseline vision (1.88 [SE, CF] ± 0.57 logMAR). The mean final vision was best in the cataract surgery group (0.57 [SE, 54/200] ± 0.67 logMAR) and worst in the vitrectomy group (1.38 [SE, 21/500] ± 1.07 logMAR) with the difference being significant (*P* = 0.001). The post-treatment improvement in final visions was significant in the group that received prior cataract surgery (*P* < 0.001) and prior vitrectomy (*P* = 0.034) after treatment.

**Table 3:** The findings and differences between different prior intraocular procedures

Surgery (n)	Cataract surgery (66)	Vitrectomy (12)	IVI (4)	*P*
**Age**(mean, SD), y	69.27 (8.04)	61.00 (13.39)	60.75 (9.54)	0.005^c*^
**Male**(n, %)	39 (59.09)	5 (41.67)	2 (50)	0.518^e^
**Duration**(mean, SD), d	3.07 (2.31)	3.33 (0.56)	6.0 (5.89)	0.918^c^
**Interval**(mean, SD), d	2.35 (1.97)	2.42 (2.94)	1.75 (0.96)	0.998^c^
**DM** (n, %)	22 (33.33)	6 (50)	2 (50)	0.463^e^
**Hypopyon** (n, %)	55 (83.33)	4 (50)	0 (0)	0.243^e^
**Vitritis** (n, %)	59 (89.39)	12(100)	4(100)	0.395^e^
**Treatment**				
VT(n, %)	55 (83.33)	12 (100)	4 (100)	0.214^e^
IVI alone(n, %)	11 (16.66)	0 (0)	0 (0)	0.214^e^
IVI total(mean, SD)	2.64 (1.30)	2.58 (0.79)	1.75 (0.96)	0.891^c^
**Culture positive**(n, %)	21 (31.8)	5 (41.67)	4 (100)	0.021^e*^
**Complications**(n,%)	14 (21.2)	3 (25)	1 (25)	0.948^e^
**Follow-up time**(mean, SD)	13.4 (17.96)	20.75 (24.96)	11.25 (16.05)	0.224^c^
**Baseline vision**(mean, SD)	1.88 (0.57)	2.09 (0.35)	2.08 (0.26)	0.414^c^
**Final vision**(mean, SD)	0.57 (0.67)	1.38 (1.07)	0.97 (0.74)	0.001^c*^
*P*	0.000^d*^	0.034^d*^	0.109^d^	

^*^P < 0.05, ^c^ANOVA, post-hoc cataract surgery and vitrectomy, ^d^Paired *t-*test; ^e^chi-squared test; n, number; %, percentage; SD, standard deviation; y, years; d, days; VT, vitrectomy; IVI, intravitreal injection; logMAR, logarithm of minimum angle of resolution

**Fig. 1:**
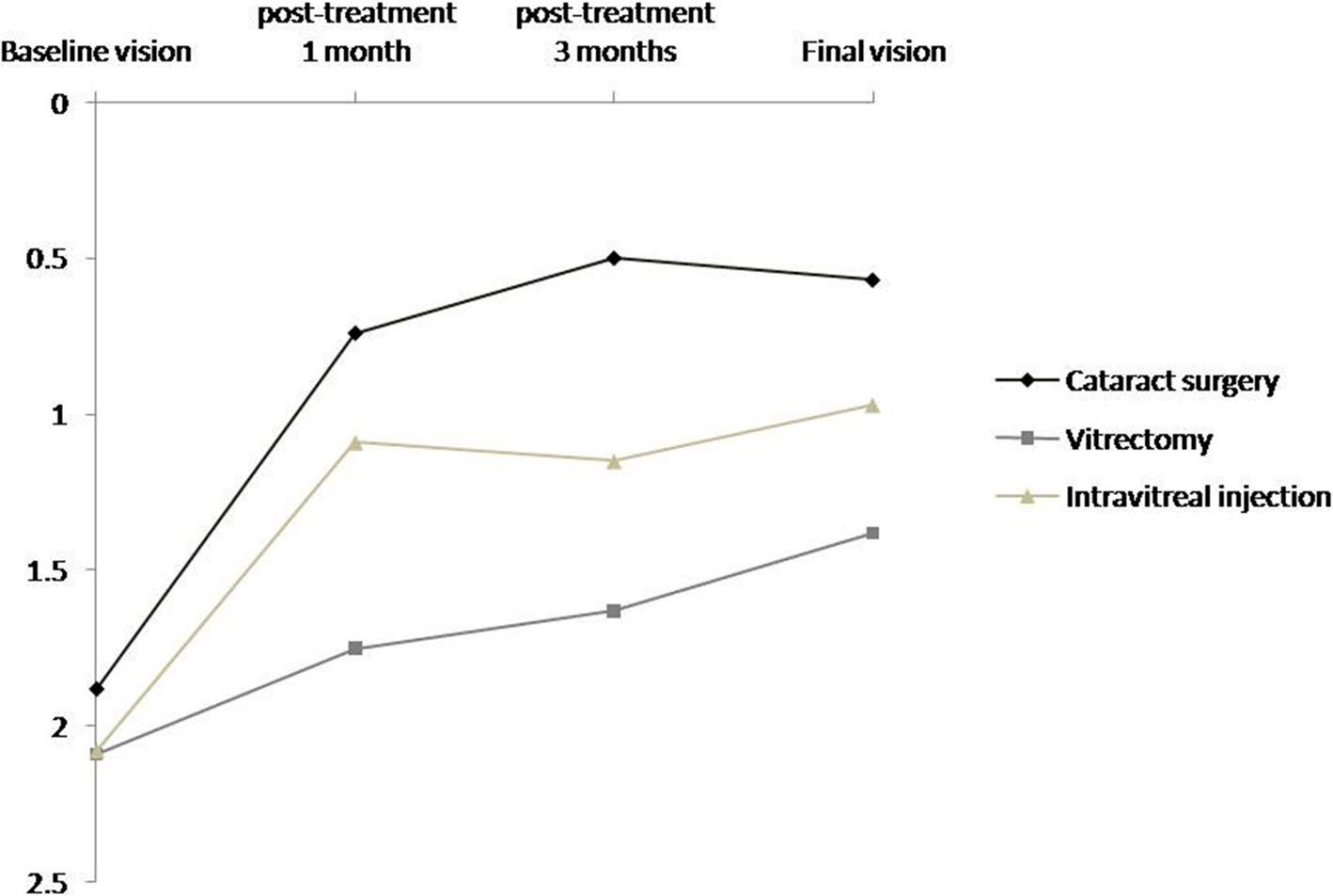
Vision changes before and after treatment in prior cataract surgery, vitrectomy, and intravitreal injection groups. The mean baseline vision in the group of prior cataract surgery is 1.88 ± 0.57 logarithm of the minimum angle of resolution (logMAR) and notably improves to 0.74 ± 0.66 logMAR at 1 month post-treatment, 0.5 ± 0.6 logMAR at 3 months post-treatment, and 0.57 ± 0.67 logMAR of final vision. In the group of prior VT, the mean baseline vision was 2.09 ± 0.35 logMAR and slowly improves to 1.75 ± 0.85 logMAR at 1 month post-treatment, 1.63 ± 0.88 logMAR at 3 months post-treatment, and 1.38 ± 1.07 logMAR of final vision. In the groups of prior IVI, the mean baseline vision is 2.08 ± 0.26 logMAR and gradually improves to 1.09 ± 0.43 logMAR at 1 month post-treatment, 1.15 ± 0.21 logMAR at 3 months post-treatment and 0.97 ± 0.74 logMAR of final vision. For the final vision after treatment of acute postoperative infectious endophthalmitis, the visual outcome is best in the cataract surgery group, second best IVI group, and third best in the vitrectomy

Figure 1 summarized the vision changes from baseline to 1 and 3 months, post-treatment and the final vision in three groups with different prior intraocular surgeries. The mean baseline vision in the group that underwent cataract surgery was 1.88 (SE, CF) ± 0.57 logMAR better than that in the vitrectomy and IVI groups with (2.09 [SE, CF to HM] ± 0.35 logMAR) and (2.08 [SE, CF to HM] ± 0.26 logMAR). The 1 month post-treatment vision in the cataract surgery group improved to 0.74 ± 0.66 logMAR, and that in the vitrectomy and IVI groups also progressed to 1.75 ± 0.85 logMAR and 1.09 ± 0.43 logMAR, respectively. The 3 month post-treatment vision in the cataract surgery group continued to improve to 0.5 ± 0.6 log MAR, which was still better than that in the vitrectomy and IVI groups (1.63 ± 0.88 logMAR and 1.15 ± 0.21 log MAR, respectively). The final vision in the cataract surgery group was maintained at 0.57 ± 0.67 logMAR while those in the vitrectomy and IVI groups continued to improve to 1.38 ± 1.07 logMAR and 0.97 ± 0.74 logMAR, respectively. For the final vision after treatment of acute postoperative infectious endophthalmitis, the visual outcome was best in the cataract surgery group, second best in the IVI group and third best in the vitrectomy group.

## Discussion

The treatment of endophthalmitis has evolved over the last several decades [12]. Before the era of vitreoretinal surgery, enucleation of the affected eye was performed [3]. In the 1970s, treatment of endophthalmitis was greatly improved by intravitreal antibiotic which is still used in many countries as the primary treatment, and have been called the “silver standard” by ESCRS guidelines [13]. In our study, 8.5% of the infected eyes had mild to moderate vitritis, and these eyes showed a better final vision of 0.12 (SE, 76/100) logMAR The infected eyes that only received IVI of antibiotics (13.30%) showed a significantly better final vision of 0.28 (SE, 105/200) logMAR than those that received vitrectomy with IVI antibiotics. Low bacterial loading and inflammation could be well controlled by the IVI of antibiotics alone, and vitritis may clear up spontaneously. Thus, the lower severity and toxicity of endophthalmitis causes less retinal damage, yielding better visual outcomes.

Later, the EVS group concluded that early vitrectomy in endophthalmitis was only beneficial to patients with baseline vision of LP or worse who had a three-fold vision improvement of achieving 20/40 vision after vitrectomy [14]. Delayed vitrectomy could improve vitreous clarity after an episode of endophthalmitis [15]. Some studies have proposed that persisting with levels of vitreous antibiotics in excess of the MICs for 3 days or more after IVI and repeating the same agents for 2–3 days would carry further risk of retinal toxicity [16, 17]. Since 1980, the success rate of PPV, defined as a final vision of 20/400 or better, has increasingly improved ranging from 42 to 73%. Therefore, complete PPV is now recommended the “gold standard” treatment of endophthalmitis [13, 18]. In our study, severe vitritis accounted for 91.6% of the cases, which showed a worse final vision of 0.76 (SE, 35/200) logMAR than those in the group of mild to moderate vitritis with a significant difference. Meanwhile, the mean baseline vision in the mild to moderate vitritis group was 0.97 (SE, 21/200) logMAR, which improved to mean final vision of 0.12 (SE, 150/200) logMAR with significance. The baseline vision in the severe vitritis group was 2.0 (SE, CF to HM) logMAR, which improved to mean final vision of 0.77 (SE, 34/200) logMAR with significance either. For mild to moderate vitritis, delayed vitrectomy is warranted when the vitreous opacity after IVI antibiotics cannot be cleared up spontaneously. For severe vitritis, early vitrectomy could reduce the large amount of bacterial loading and toxicity in the vitreous, avoiding further retinal damage. For treatment strategies of the VT combined with IVI and IVI alone groups, the baseline vision in the group of VT combined with IVI was 1.95 (SE, CF to HM) logMAR and improved to 0.78 (SE, 33/200) logMAR with significance, while the baseline vision in the group of IVI alone was 1.69 (SE, 4/200) logMAR and improved to 0.28 (SE, 106/200) logMAR with significance. These results suggested that early vitrectomy for severe vitritis and IVI alone for mild to moderate vitritis could both lead to final vision improvements. Besides, the mean amounts of IVI antibiotics were 2.59 ± 1.23 injections. For the group of VT combined with IVI, 1–2 more injections would control infection. For the IVI antibiotics alone group, administering 2–3 injections could inhibit infection. Moreover, the amounts of IVI antibiotics were significantly associated with final visions. For severe endophthalmitis, increasing number of IVI antibiotics would be necessary to control. Some studies found that even after successful treatment of endophthalmitis, perifoveal retinal inner layers atrophy [19] and decreasing choroidal thickness [20] associated with visual impairment. Thus, both toxicity of antibiotics and bacterial infection can affect the final vision.

In our study of acute infectious postoperative endophthalmitis, the related factors affected the final visual outcomes included pre-treatment factors of diabetes, hemodialysis, baseline vision, severity of vitritis; treatment factors of treatment strategies and the amount of IVI antibiotics, and post-treatment complications. The group with post-treatment complications had a worse final vision of 1.44 (SE, 18/500) logMAR than that without complications with 0.5 (SE, 63/200) logMAR. About EVS data, 8.3% of the patients developed RD after initial treatment [21] whereas the incidence of RD was 9.75% in our study, and didn’t significantly affect the final vision. For the group of prior cataract surgery with complications of RD later, the final vision would be not inferior to the mean final vision of the group with complications if retina attached finally. For the prior vitrectomy surgery with complications of RD later group, the previous retinal disease such as PDR would deteriorate the final visions. For recurrent RD after complications of RD, the groups of prior cataract surgery and vitrectomy both had a worse final vision in the group with complications regardless of retinal attachment at last.

Regarding the duration between prior surgery to oneset of symptoms, the prior IVI group showed a relatively late-onset attack compared to the prior vitrectomy group, while the prior cataract surgery group showed a relatively acute onset attack. This was due to the relatively poor vision of patients who required IVI and vitrectomy since early vision deterioration could not be detected. Additionally, bacteria taken directly into the vitreous cavity after performing surgical procedures revealed less anterior inflammatory reaction which could be ignored in early phase. This could be explained by the higher rate of vitritis which showed 100% in prior vitrectomy and IVI groups and the relatively higher rate of positive culture results showed 41.67% in prior vitrectomy group and 100% in prior IVI group. In contrast, patients experienced endophthalmitis after prior cataract surgeries because pathogens entered into the anterior chamber to induce inflammation or infection, which was detected early. Concurrently, the prior cataract surgery group showed relatively lower rates of vitritis (89.39%) and positive culture results (31.8%). Regarding treatment strategies, all patients in the prior vitrectomy and IVI groups subsequently received vitrectomy combined with IVI antibiotics rather than IVI antibiotics alone. The best final post-treatment vision was found in the prior cataract surgery group, followed by the prior IVI group, while the worst final post-treatment vision was found in the prior vitrectomy group.

Oshima et al. proposed the final visual outcomes were much better in the group of 23-G or 25-G transconjunctival microincision than that after conventional 20-G vitrectomy [22]. However, we found no statistically significant difference in final vision between conventional 20-G and 23-G transconjunctival microincision. The visual outcomes are generally related to organism virulence [23] and past severity of retinopathy. Park et al. reported the incidence of 0.022% (2/9159) for bevacizumab of IVI-induced endophthalmitis with staphylococcus species, and while one eye still maintained vision after treatment, the other developed phthisis [24]. In our study, three eyes were bevacizumab IVI-induced endophthalmitis and one was ranibizumab IVI-induced. The culture results showed staphylococcus species in three eyes and mixed flora in one eye. The mean final vision showed 0.97 (SE, 53/500) logMAR with foveal scars in two eyes and macular ischemia in one eye, and no lesion in one eye, related to previous maculopathy and retinopathy.

The potential limitations were small size in the prior vitrectomy and IVI groups, and the retrospective, nonrandomized study design.

## Conclusions

Factors related to a poor final visual prognosis were diabetes and renal disease, poor baseline visions, severe vitritis that occlude the disc and retinal vessels, vitrectomy with IVI of antibiotics, amounts of IVI antibiotics, and post-treatment complications. For visual outcomes, patients who had undergone prior cataract surgeries showed better visual outcomes than those who had undergone prior vitrectomy or IVI.

## Data Availability

This study is based in part on data from the Department of Medical Education and Research and Research Center of Medical Informatics in Kaohsiung Veterans General Hospital. The datasets used and/or analyzed during the current study are available from the corresponding author, Tsung-Tien Wu, on reasonable request.

## Abbreviations

IVI: Intravitreal injection
IOL: Intraocular lens
PPV: Pars plana vitrectomy
ESCRS: European Society of Cataract and Refractive Surgeons
IRIS: Intelligent Research in Sight
G: Gauge
anti-VEGF: Anti-vascular endothelial growth factors
BCVA: Best-corrected visual acuity
log MAR: logarithm of the minimal angle of resolution
DM: Diabetic mellitus
HTN: Hypertension
IOP: Intraocular pressure
RD: Retinal detachment
RRD: Rhegmatogenous retinal detachment
VMT: Vitreomacular traction
PDR: Proliferative diabetic retinopathy
VH: Vitreous hemorrhage
SE: Snellen Equivalent
CF: Counting finer
HM: Hand motion
EVS: Endophthalmitis Vitrectomy Study
